# An erroneous opinion on a cause of death in a forensic autopsy: a case report

**DOI:** 10.4314/ahs.v17i4.36

**Published:** 2017-12

**Authors:** Meel Banwari

**Affiliations:** Department Laboratory Medicine and Pathology, Faculty of Health Sciences, Walter Sisulu University, Mthatha 5117 Tel: 047 502 2961, Cell: 0822007460

**Keywords:** Erroneous opinion, forensic autopsy

## Abstract

**Background:**

The quality of autopsies is always questioned in courts, especially in developing countries. Wrong decisions or misjudgments are undesirable in medicine, but they are very dangerous in forensic medicine. If a wrong opinion is given, either a culprit can be acquitted or an innocent person can be sentenced. Therefore, an expert opinion is always required before the announcement of a judgment.

**Objective:**

To highlight the problem of accuracy in determining the cause of death in forensic autopsy.

**Case history:**

A 19- year old young adult male (Mr E), who had a history of alcohol abuse, was brought to a hospital casualty department by police, on an allegation of theft. He was unconscious and died within two hours of arrival. A post-mortem report was requested by a private attorney for an expert opinion. A post-mortem examination was conducted and multiple superficial injuries were recorded on his body. Head injury was given as a cause of death. The author seeks to critically analyze the post-mortem findings in relation to the cause and manner of death.

**Conclusion:**

An erroneous opinion was reached regarding cause and manner of death in this autopsy report.

## Introduction

The importance of an autopsy in establishing the cause of death accurately is important in natural deaths, but it is even more important in the event of unnatural deaths. Often the cause of death looks quite obvious, especially in mechanical injuries. To determine the underlying or immediate cause of death, the manner of death and conditions contributing to death can be problematic.^1^ The cause of death is that which produces an effect. It is a combination of circumstances that must precede and that invariably result in an effect.^2^

The underlying cause of death is the disease or injury that initiated the train of morbid events leading directly to death.^3^ This underlying cause of death must always be etiologically specific and antecedent to all other causes with respect to a pathological relationship.^4^ A study carried out by Hirsch showed that without an underlying cause, death would not have occurred.^5^ The presumed cause of death was found to be wrong in 28% of cases.^6^

Alcohol should be considered a dangerous drug, considering the small difference between a harmless social drink and a fatal dose.^7^ Alcohol and traumatic brain injury are closely related in up to two-thirds of people who die from that cause.^8^ Sub-dural hemorrhages are especially common in alcoholics because of frequent unprotected falls.^9^ A small self-limiting sub-dural hemorrhage may remain asymptomatic and would be an incidental finding at autopsy.^9^ The question of the blood alcohol concentration necessary to cause death is not easy to answer and widely different opinions exist among specialists in forensic toxicology and legal medicine.^10^ Forensic pathologists are familiar with alcohol abusers who are found dead and that whose cause of death cannot be ascertained.^11^

The purpose of this article is to provide information on the accuracy of cause of death in medico-legal autopsies, and to raise the awareness of law enforcement authorities that they have to be careful in making decisions in such cases.

## Case history

A 19-year-old young adult male, who had a positive history of (abusively) drinking alcohol, was alleged to have stolen cell phone in a gathering. The incident was reported to police. They took him to hospital, as he was under the influence of alcohol. On arrival at hospital, his blood pressure was 237/135, pulse 72 per minute, and glucose 11 mmol. His temperature was 36 degrees centigrade and his respiratory rate 24 breaths per minute. Blood oxygen saturation was 99%, hemoglobin was 10 grams per deciliter. He was unable to stand on his feet and could not pass urine. His general condition was poor, he was unresponsive and the Glasgow Comma Scale was 3/15.There was bleeding from a laceration on his cheek. There were multiple bruises on his forehead and right orbit. Both legs were bruised and swollen, with abrasions on both knees. His condition deteriorated fast and he was confirmed dead within two hours of arrival.

On postmortem, there were peri-orbital odema both sides, bruised and edematous tip of nose, outer left and right eye. Multiple irregular bruised abrasions on forehead were found. Bleeding from left ear was also recorded. A stitched wound about 1cm in size was mentioned on left check. There were multiple abrasions on front of chest, front of knees, right foot, elbow and ankle. The scalp and skull were reported intact. A thin layer of subdural hemorrhage was found on the surface of brain. Brain was mildly edematous. Stomach was empty, and there was no food material in trachea and bronchi. Lungs, liver pancreas, spleen and kidneys were congested.

## Discussion

Courts tend to rely heavily on the post-mortem report, which must be conducted by an expert in this field. It reveals the full truth that could not be picked up by clinicians or radiologists in their investigations. Therefore it is considered a gold standard in certifications of death. There are very few trained specialists in forensic pathology in South Africa, despite the fact that there is a high rate of non-natural deaths. Most autopsies are carried out by medical practitioners who have limited knowledge in the field.

All injuries must be documented in a post-mortem report. Ideally, the study of a pattern of injury, along with the history of causation, should give enough information to a pathologist regarding the manner of death. Mr E's postmortem report indicated the injuries were on the frontal aspect of the body only. The pattern is indicative of an unsupported accidental fall. They were mainly on the face, chest, knees, right foot, and right ankle and the back of the left elbow. There were no injuries on the palms of the hands. In accidental falls, a normal sober individual generally uses the hands to protect himself from facial injuries. This type of pattern of injury suggests that Mr E had not used his hands to protect himself at least against facial injuries. There were only two possibilities. The victim was either not in a position to protect himself, due to drunkenness, or his hands were tied, so that he was unable to use them. Frequent unprotected falls are common among alcoholics.^9^ Most of his injuries were superficial (Mr E): multiple contusions, abrasions and a laceration (stitched). It supports the theory of accidental falls.

On post-mortem, only superficial injuries were recorded on the forehead of the victim, without any scalp injuries and fracture of frontal bones. It means that the impact was not a heavy one on the forehead. The brain is a tightly packed structure in the skull cavity with anterior, middle and posterior cranial fossa. The chances of brain damage or duramater movements are minimal in such a packed structure when force is applied to the frontal bone. Moreover, the occipital lobe is supported by the spinal cord, which does not allow any movement of the posterior. Sub-dural hemorrhage is typically due to disruption of veins that bridge the brain to the venous dural sinuses. The hemorrhage is presumed to arise from acceleration/deceleration forces that cause the dura to move in relation to the cerebral hemispheres.^12^ Because the blood is under very low pressure (from veins) the hemorrhage tends to collect slowly, causing signs and symptoms that develop over days to months.^12^ There were no signs of increased intracranial pressure (e.g. vomiting) in hospital, nor had any become evident on autopsy examination of the brain, such as tonsillar herniation. Head injuries alone could not explain the mechanism of death. Patients may not die of their disease, but rather of complications of their disease.^13^ Mr E had a mild head injury, which was not enough to cause complications. A mechanism is a physiological derangement or a biochemical disturbance produced by a cause of death.^1^ There is no description in the autopsy report of any physiological or biochemical disturbance involved in Mr E's death.

Mr E had died soon after being admitted to hospital (<2 hours), and therefore not enough sub-dural hemorrhage had occurred to increase the pressure inside the cranium. This was confirmed by the autopsy report; only a thin layer of subdural hemorrhage was found on the surface of the brain. Moreover, it was not associated with any other hemorrhage, such as epidural, sub-arachnoid and intra-cerebral hemorrhage. Solitary acute sub-dural hemorrhages are seen less frequently. They are usually associated with head trauma severe enough to cause skull fracture and cerebral contusion or laceration.^12^

The finding of mild edema of the brain is also controversial in the circumstances, as this normally occurs during each and every death. When a person dies, cells die, which results in swelling of those cells, leading to mild edema. In the case of brain edema it is also called cerebral edema or swelling of the brain, which is a non-specific finding in the death of an individual. Diffuse axonal injury is a relatively rare type of brain trauma. Corpus callosum lesions are most closely associated with diffuse axonal injury. 14 In this case the cause of death had been recorded as head injury, but no scalp injury was recorded in the report. The skull was intact. Bleeding from the left ear was mentioned, but the source of injury was again not described in the post-mortem report.

A retrospective survey was carried out of all cases where death had resulted from the complications of excessive alcohol consumption in order to determine what constituted a lethal level of alcohol intake. The cases fell into two groups, one where there was an element of asphyxia, which had complicated the effects of alcohol and led to death, and another where death was entirely due to uncomplicated alcohol poisoning.^10^ It is suggested that death can occur from uncomplicated alcohol poisoning when there is a postmortem blood alcohol level above 250 mg per 100 ml.^10^ This could probably have happened in Mr E's case, as he had consumed an excessive amount of alcohol, although blood was not collected at the time of autopsy for estimation of alcohol. This is an act of serious omission on the part of the medical officer who conducted the postmortem.

A recent study (2014) showed that individuals intoxicated by alcohol suffer higher mortality. In total 23,983 patients with severe traumatic brain injury were evaluated, of which 22.8% tested positive for alcohol. Alcohol-positive patients were more likely to suffer complications and higher mortality rates.^15^ These individuals are more vulnerable to falls, resulting in fatal injuries.^9^ The majority of poisoning deaths occur among persons who have either deliberately or accidently taken an overdose of a poisonous substance such as alcohol. A typical example is Mr E, who had probably consumed excessive alcohol, sustained minor injuries and died.

No lethal head injury was recorded in the post-mortem findings. There was only one laceration (stitched) of about 1 centimeter long on the face, along with multiple bruises and abrasions on the body. These injuries (Mr E) were not sufficient to cause death in the course of nature. It is difficult to ascertain what had played a major role in causing the death of Mr E. What condition initiated the sequence of events leading to death? In the absence of another acceptable explanation, alcohol intoxication initiated the sequence of events leading to injuries on body of Mr E. Sudden and unexpected death is another problem that occasionally raises difficult questions in certification of the death of an individual such as Mr. E. Physiological or biochemical derangement may remain unexplained on autopsy, such as a blood glucose level of 11 mmol, but surprisingly the stomach proved to be empty on autopsy. The medical officer who conducted the autopsy probably presumed that the cause of death was head injury, which was not substantiated by any supportive evidence in his post-mortem report. The presumed cause of death was found to be completely wrong in 28% of cases in a study undertaken in 2003.^6^

## Conclusion

An erroneous opinion was reached regarding the cause of death. It is difficult to implicate head injury alone as a cause of death where there was a positive history of alcoholic intoxication. It is reasonable to accept that alcohol has contributed, directly or indirectly, in causing this death. It is wrong to presume the manner of death as homicide, especially without any patho-physiologic derangement sustained through a lethal injury and its consequences.

## Ethical consideration

The name of the victim and medical officer were not revealed. The name and signature of the medical officer is covered with black ink in the sketch diagram.
